# Recurrent bladder urothelial carcinoma complicated with primary bladder large cell neuroendocrine carcinoma: a case report and literature review

**DOI:** 10.3389/fonc.2024.1369649

**Published:** 2024-08-02

**Authors:** Jiarui Cui, Qing Zhao, Chunhong Yu, Pengfei Ma, Shoubin Li

**Affiliations:** ^1^ North China University of Science and Technology, Tangshan, Hebei, China; ^2^ Department of Urology, Hebei General Hospital, Shijiazhuang, Hebei, China; ^3^ Department of Urology, Kailuan General Hospital, Tangshan, Hebei, China; ^4^ Department of Health Examination Center, Hebei General Hospital, Shijiazhuang, Hebei, China

**Keywords:** bladder tumor, LCNEC, diagnosis, treatment, prognosis

## Abstract

**Objective:**

To improve the understanding, diagnosis and treatment of bladder large cell neuroendocrine carcinoma (LCNEC).

**Methods:**

A clinical case of bladder LCNEC admitted to our hospital was reported. The epidemiology, prognosis, diagnosis and treatment methods of large cell neuroendocrine carcinoma were reviewed. The diagnosis and treatment status and prognosis were discussed based on the literature.

**Results:**

The female patient was admitted to hospital for “more than 4 years after TURBT and intermittent hematuria for more than 2 years”. She was diagnosed as recurrent bladder cancer and underwent “radical cystotomy + hysterectomy”. The postoperative pathological findings were high-grade urothelial carcinoma of the bladder neck and large cell neuroendocrine carcinoma of the bladder. The patient recovered well after surgery, but refused radiotherapy and chemotherapy and is still under close follow-up.

**Conclusion:**

Bladder LCNEC is clinically rare, has unique pathological features, is more aggressive than traditional urothelial carcinoma, and has a poor prognosis. Surgery, chemotherapy and radiotherapy should be combined with multi-mode treatment.

## 1 Introduction

Primary bladder neuroendocrine carcinoma is very rare, accounting for less than 1% of bladder tumors (1), among which large cell neuroendocrine carcinoma (LCNEC) is even rarer, with only about 50 cases reported so far (2). It is characterized by poor differentiation and strong aggressiveness (2), and the most common symptom is gross hematuria (3).Because the disease is very rare, there is no relevant diagnosis and treatment guidelines and expert consensus. We report a case of recurrent bladder urothelial carcinoma with LCNEC in order to improve the understanding of this disease and improve the level of diagnosis and treatment.

## 2 Case presentation

**Figure 1:**
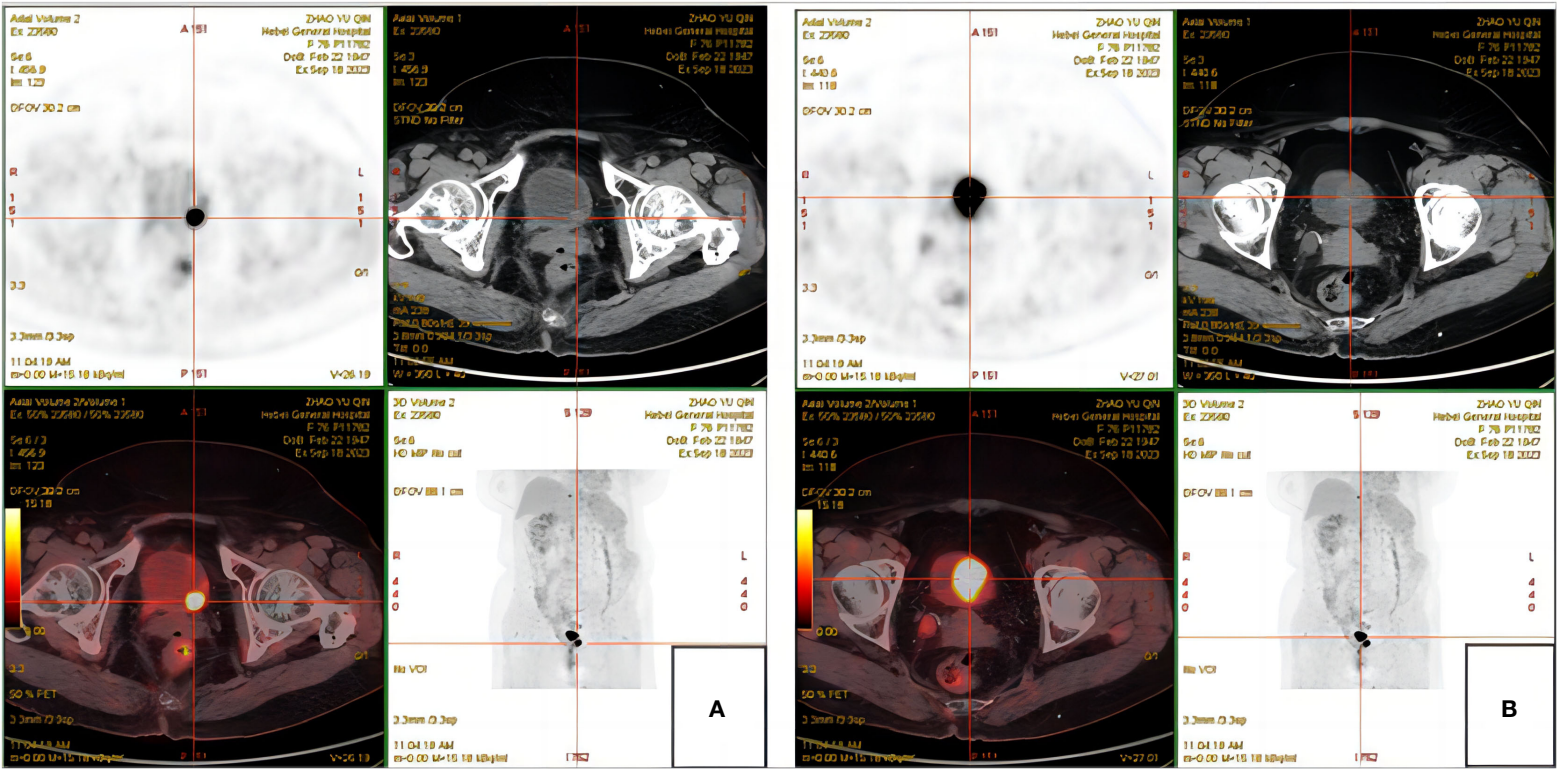
PET-CT. **(A)** High metabolic wall occupation in the left posterior wall of the bladder. **(B)** High metabolic wall occupation near the upper margin of the bladder body.

**Figure 2:**
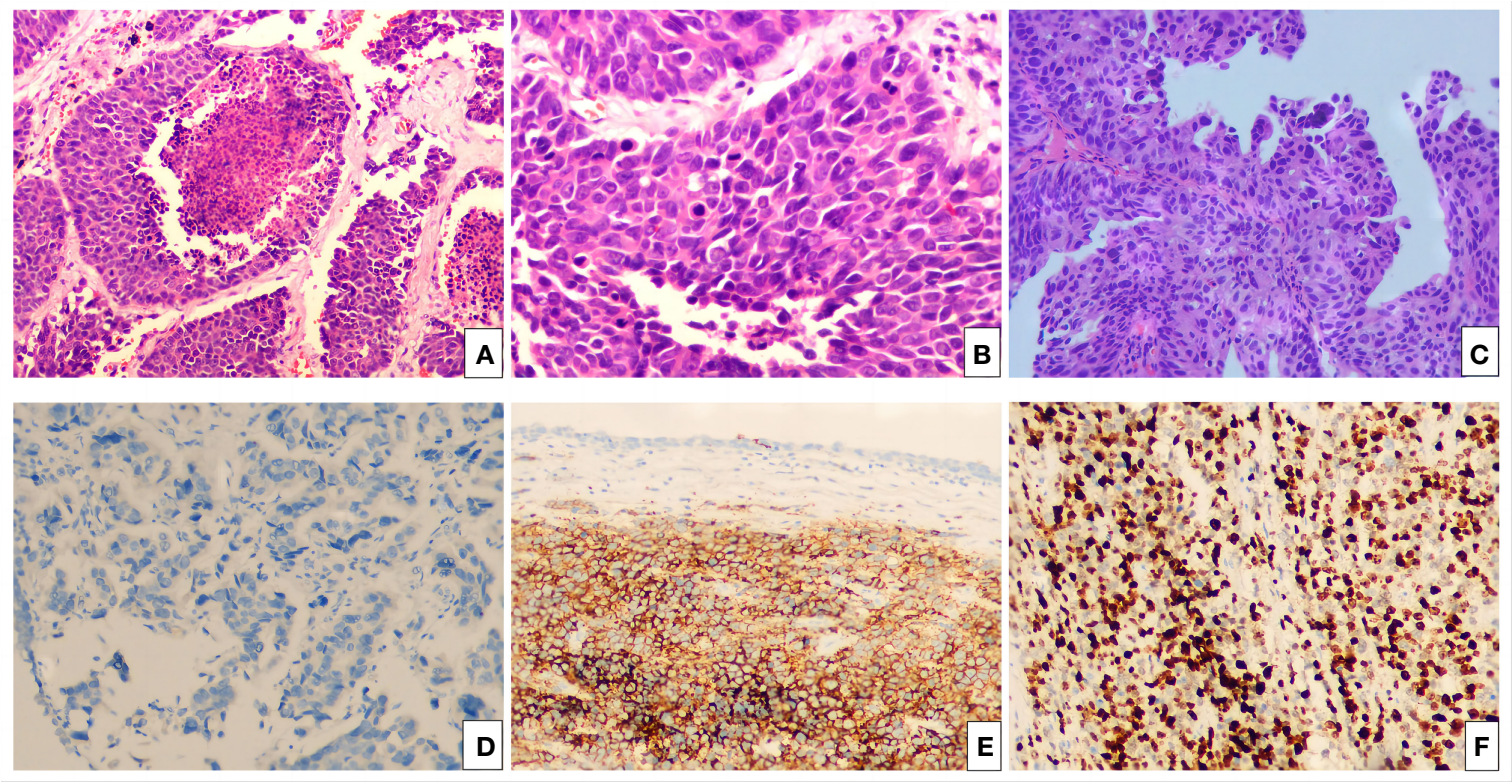
The hematoxylin-eosin (HE) and immunohistochemical pictures. **(A)** Bladder LCNEC cells arranged in an organoid nest growth pattern. (H&E staining, ×100). **(B)** Bladder LCNEC cells are large, the nucleus is large, the nucleus is polymorphic, and the nucleolus is prominent (H&E staining, ×400). **(C)** High-grade urothelial carcinoma of the bladder neck (H&E staining, ×100). Immunohistochemistry: Syn (+) **(D)**, CgA (+) **(E)**, Ki-67 (about 70%+) **(F)**.

**Figure 3:**
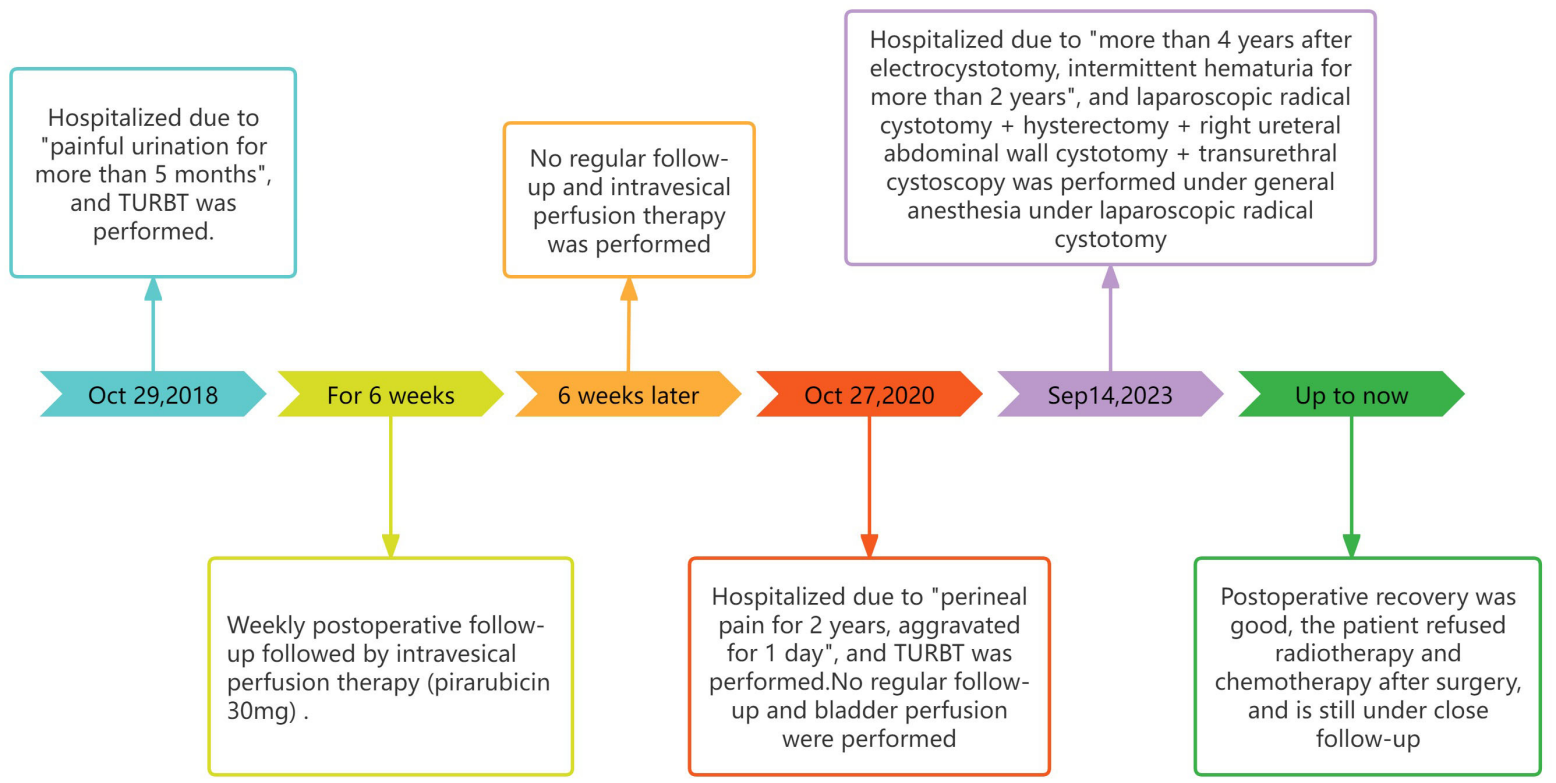
Treatment timeline of this case.

The patient, a 76-year-old female, was admitted to hospital for the first time on October 29, 2018, mainly due to “painful urination for more than 5 months”. Cystoscopy indicated bladder tumor, and transurethral resection of bladder tumor (TURBT) was performed. The postoperative pathological results showed as follows: Severe urothelial dysplasia, considering cancer”, weekly postoperative follow-up followed by intravesical perfusion therapy (pirarubicin 30mg) for 6 weeks,6 weeks later, no regular follow-up and intravesical perfusion therapy was performed. The patient was readmitted for the second time on October 27, 2020, due to “perineal pain for 2 years, aggravated for 1 day”. Cystoscopy was performed to obtain pathological findings: (bladder neck) biopsy tissue: high-grade urothelial carcinoma. Transurethral resection of bladder tumor was performed under general anesthesia. Postoperative pathological findings showed that urothelial dysplasia was considered as carcinoma. Since the patient felt that the perineal pain symptoms basically disappeared after surgery, we emphasized the necessity of regular follow-up and bladder perfusion therapy, but the patient still did not pay attention to it, and no regular follow-up and bladder perfusion therapy were performed after surgery. The patient visited the doctor on September 14, 2023, this time because of “more than 4 years after TURBT, intermittent hematuria for more than 2 years”, accompanied by urinary pain. The preoperative urine routine tests demonstrate a significant abundance of red and white blood cells (RBC: 51.0ul;WBC: 52.6ul). Preoperative ureteral computed tomography (CT) scan showed irregular high-density shadow in the bladder, the size of about 27 × 30mm, the enhanced scan showed uneven enhancement, and the boundary between the lesion and the bladder wall was unclear. Imaging diagnosis: “Possibility of bladder cancer recurrence”. PET-CT indicated: “The bladder body has a high metabolic wall occupying the near upper margin and the left posterior wall, which is consistent with the recurrence of bladder cancer combined with the medical history, and no clear signs of metastasis and recurrence were found” (**Figure 1**). The patient had a history of diabetes for 9 years and had undergone left nephrectomy for renal tuberculosis more than 40 years ago. She had no history of other diseases or family history. After completing the preoperative preparation, radical cystotomy + hysterectomy + right uretero-cutaneostomy + transurethral cystoscopy was performed under general anesthesia (**Figure 2**):”1. (bladder) combined with immunohistochemical staining supports high-grade neuroendocrine carcinoma, considered to be a large cell neuroendocrine carcinoma, invading the muscle proper of the bladder wall, showing intravascular cancer embolus(CD31,CD34,D2-40) and nerve infiltration. Immunohistochemical staining: Ki-67 (about 70%+), P53 (+++), CK7 (-), CK20 (-), P63 (focal +), GATA-3 (-), CK5/6 (-), P40 (-), P16 (+), E-cadherin (focal +), Syn (+), CgA (+), CD56 (+).2. High-grade urothelial carcinoma (neck of bladder), invading the muscularis propria of the tube wall. Immunohistochemical staining: Syn (-), CgA (-), CD56 (-), P63 (-), CK7 (+), P40 (-), GATA-3 (+), Ki-67 (about 30%+), CK20 (+). Incision margin of urethra negative. The individual was eventually diagnosed with primary bladder LCNEC with urothelial carcinoma (pT_2_N_0_M_0_). Given the rarity of this disease and the lack of clear treatment guidelines, chemotherapy with cisplatin and etoposide is planned to prevent metastasis and recurrence after discussion by a multidisciplinary team. However, due to her own reasons, the patient refused radiotherapy and chemotherapy after surgery. The patient recovered well after surgery, and no metastasis or recurrence was observed during the follow-up 8 months after surgery, which is still under close follow-up. The treatment timeline for this patient is summarized in **Figure 3**.

## 3 Discussion and conclusion

Neuroendocrine neoplasms (NENs) are a class of rare neoplasms that originate from peptides and neuroendocrine cells, have neuroendocrine differentiation and express neuroendocrine markers, and can occur throughout the body, most commonly in the respiratory tract and gastrointestinal tract (4).Bladder neuroendocrine tumors are rare. According to the classification of WHO in 2022, primary bladder neuroendocrine tumors can be subdivided into five categories: small cell neuroendocrine carcinoma (SCNEC), large cell neuroendocrine carcinoma (LCNEC), well-differentiated neuroendocrine tumor (NETs), mixed neuroendocrine neoplasms and paraganglioma (5). LCNEC is the least common subtype, and relevant data are scarce, mainly described in case reports or small case series. Since Abenoza et al. first discovered bladder LCNEC more than 30 years ago (6), only about 50 cases have been reported up to now. Neuroendocrine carcinomas of the urinary bladder (NECB) etiology is not yet clear, the current theory is widely accepted by urinary tract intraepithelial developed from stem cells or induced pluripotent stem cells, according to this theory, the development of multicentric cancer cells is accompanied by changes in the progression of bladder cancer, which have been shown to be closely related to smoking (7), and another hypothesis is that neuroendocrine cells originate from the epithelium remaining in the urachus in the bladder (8). Most of the cases reported to date present as co-existing with other tumor types, the most common being mixed urothelial carcinoma, followed by adenocarcinoma, squamous cell carcinoma, and sarcomatoid urothelial carcinoma. Bladder LCNEC is characterized by poor differentiation, strong invasiveness, high metastasis potential and poor prognosis, and is usually found in the late or metastatic stage (2). And it tends to affect men, with about 80 percent of diagnosed cases occurring in men. The average age at diagnosis is close to 60 years (1). The most common symptom is gross hematuria (3), other symptoms include difficulty in urination, painful urination, frequent urination, or local abdominal or pelvic pain (1), it may even be asymptomatic.

The lack of specific clinical symptoms and laboratory findings of bladder LCNEC requires cystoscopic biopsy and pathology. The initial radiographic evaluation of NECB, including LCNEC, is usually by CT. The study results of Boyer et al. (9)showed that NECB was generally presented as an invasive and isolated large bladder mass, accompanied by varying degrees of necrosis, calcification and strengthening, with common diffuse bladder wall thickening and extending to the peribladder fat and surrounding structures. The morphological diagnostic criteria of bladder LCNEC are the same as those of lung, kidney and ureter LCNEC (10), and LCNEC is generally considered to have the following morphological characteristics (11):1. Tumor cells were arranged in sheet, palisade, trabecular or organoid nest growth pattern.2. Single cells are large, polygonal, abundant in cytoplasm and low in cytoplasmic ratio.3. The nuclei are polymorphic, often large, oval, with coarse chromatin, granular or vesicular, often protruding nucleoli, and occasionally exotic cells can be seen. In addition, necrosis, frequent apoptotic bodies, active mitotic activity (10 or more mitosis per 10HFP), and specific growth patterns (rosette or pinnate) are more common in bladder LCNEC than in bladder SCNEC (3, 12).In addition, the histological features and immunohistochemical positive markers of bladder LCNEC showed neuroendocrine differentiation (pheochromogranin, synaptic growth protein, neuron-specific enolase and thyroid transcription factor-1) (12), and expressed synaptic growth protein, pheochromogranin, CD56 and epithelial markers. Namely pancytokeratin, CAM5.2 and EMA. The sensitivity and specificity of the above immunohistochemical staining were 96% and 100%, respectively, for differentiating urothelial carcinoma (UC). It has been reported in the literature that CD56, synaptic growth protein and chromogranin are expressed in most cases (100%, 92.6% and 85.2%, respectively) (13); however, it has also been reported that chromogranin A staining is less sensitive to large cell neuroendocrine carcinoma than to small cell carcinoma (40% and 80%, respectively) (10, 14). In addition, Ki67 index >40% has been shown to have 80% sensitivity and 86% specificity in distinguishing between LCNEC and UC (15). Differential diagnosis of primary bladder LCNEC includes metastatic LCNEC(most commonly derived from the lung and intestine), local extensions of poorly differentiated prostate cancer, high-grade urothelial carcinoma, SCNEC, poorly differentiated transitional cell carcinoma, and some types of lymphoma of the bladder (16). Immunohistochemistry can be used to distinguish LCNEC from non-neuroendocrine lesions. Because bladder LCNEC may come directly from the prostate, it is also important to evaluate the history of prostate cancer (17). In addition, clinicopathological and imaging correlations play a key role in the diagnosis of metastatic LCNEC.OSHIRO et al. (18) believed that urine cytology could be used as one of the diagnostic methods for early detection. However, diagnosis of bladder LCNEC by conventional urine cytology alone is difficult because of the low sustainability of tumor cells in the urine and their susceptibility to degeneration in the urine.

Because bladder LCNEC is a rare disease, there are no treatment guidelines or expert consensus. Wang et al. (12)reported that the median survival of 8 non-metastatic patients who did not receive neoadjuvant chemotherapy was only 8.9 months, while in the study of Niu et al. (19), the median survival of 5 patients who only received cystectomy was only 7 months. In the study of Xia et al. (20), the median survival of 26 patients receiving multimodal therapy was 22 months, while the median survival of 13 patients receiving monotherapy was 10 months. The most commonly recommended multimodal therapy is the combination of chemotherapy, radiotherapy and radical or partial surgery (21).Multiple studies have shown that patients with bladder neuroendocrine carcinoma who receive cystectomy + chemotherapy + radiotherapy have a longer survival (19).No matter adjuvant therapy or neoadjuvant therapy, platinum-based neoadjuvant chemotherapy is the treatment choice (19, 22, 23). Bhatt et al. (22) reported that platinum-based neoadjuvant chemotherapy can improve the survival rate of neuroendocrine bladder cancer, and cystectomy or TURBT combined with radiotherapy are recommended for those who are not suitable for chemotherapy. Whether radical surgery is preferable to the bladder-preserving approach remains controversial. In one of Luzzago ‘s (24)population-based researches, they found thatPC (partial cystectomy) instead of RC(radical cystotomy) in non-UCUB (variant histology bladder cancer patients, included adenocarcinoma, squamous carcinoma, neuroendocrine carcinoma, other 70 histological subtypes) patients does not undermine survival. Therefore, they conclude that the excess CSM (cancer-specific mortality) is unrelated to cystectomy type, but originates from tumor biology. Neither the retrospective study by Sroussi et al. (25) nor the systematic review by Xia et al. (20) found any benefit from radical cystectomy. However, in a research by Deuker (26) involving 37,528 T1N0M0 bladder cancer patients shows that, RC improves survival in patients with stage T1 VHBC(variational histological bladder cancer) such as SqCC(squamous cell carcinoma) and neuroendocrine carcinoma of the urinary bladder. In 249 patients with bladder neuroendocrine cancer, no cancer-related deaths were observed in 26 patients treated with RC, compared to 50/223 (22.4%) in patients not treated with RC. Besides, the five-year CSS (cancer specific survival, no death attributable to bladder neuroendocrine cancer) rate in T1 neuroendocrine bladder neuroendocrine cancer patients was 100% after RC vs. 66.8% if no RC was performed. Niu et al. (19) found that the subgroup of cystectomy +CT+RT combined treatment had the best overall prognosis. It is generally accepted that multimodal therapy should be preferred for LCNEC. It is generally believed that immunotherapy (such as intravesical BCG treatment) is not recommended as a treatment for LCNEC, but BCG treatment may be considered as appropriate because the tumor may have uroepithelial carcinoma components (27). In addition, Sun et al. (2) reported a case of the urinary bladder treated by radical cystectomy combined with toripalimab immunotherapy, achieving sustained remission, which provides a new idea for the treatment of LCNEC. By studying the genome map, van (28)et al. revealed potential therapeutic targets based on almost half of the patients with operable (and reactive) somatic aberrations in their genomes, such as TP53, CDKN1B, KRAS, etc., which may help improve treatment strategies for neuroendocrine tumors, including bladder LCNEC. Pederzoli et al. (29)summarized the current understanding of major gene fusion in urogenital malignancies and emphasized their potential use as targets of precision medicine methods. They believe that with the continuous development of this field, more new strategies for fusion gene targeted therapy of urogenital cancers, including bladder LCNEC, will be provided in the future. At present, anti-hormone endocrine therapy by inhibiting AR(androgen receptors) or ER(estrogen receptors) has become one of the important treatment methods for hormone-dependent tumors, Wucherpfennig (30)et al. ‘s research found that 22%(4/18) of patients with SCNEC were AR-positive, and they suggested that endocrine therapy such as anti-AR therapy could be applied to bladder SCNEC, and might address a significant proportion of patients. Bladder SCNEC and LCNEC are both endocrine related tumors, so endocrine therapy can also be considered as one of the treatment methods for LCNEC, but this still needs to be further studied.

Extrapulmonary Neuroendocrine carcinomas such as LCNEC are characterized by a very low incidence, aggressive behavior, frequent presentation at later stages, high recurrence rate, and hence poor prognosis (10). Claps (31) analyzed a multi-institutional cohort of 1082 patients treated with RC for urothelial BC, the research showed that more than 25% of patients harbored VHs (variant histologies) at time of RC. Compared to pure UC, clear-cell, plasmacytoid, small-cell and sarcomatoid VHs were associated with worse prognoses while lymphoepithelioma-like VH was characterized by a prognosis benefit. In the center of a involved more than 2570 patients with bladder cancer experiment at 36 institutions, Matsuda (32) analyses the impact of histological discordance of subtypes or DD (divergent differentiation)in specimens from TURBT and RC on the outcome of the patients with bladder cancer receiving RC. The study demonstrated that the presence of subtype/DD in RC specimens but not in TURBT specimens had a poor prognostic impact, and poorer survival was revealed in cases in which subtype/DD or non-UC. Accurate pathological diagnosis is very important for the treatment and prognosis of patients. In addition, regular follow-up and monitoring of patients is also very important. At present, bladder LCNEC monitoring methods are the same as other urothelial cell carcinomas (23). Cystoscopy is especially important and must be performed every 3 months. Because this tumor is easy to metastasize under the microscope, distant metastasis often occurs at the time of diagnosis or in the later stages of the disease, in which lymph nodes, liver and lung are the most common sites of metastasis (3, 20), in addition, brain and skin metastasis have also been reported (3).Therefore, radiological examination is also very necessary.

In summary, bladder LCNEC is a rare malignant tumor with strong aggressiveness and poor prognosis. Early diagnosis and comprehensive treatment are very important. The current diagnosis is mainly based on pathology, and multi-mode treatment is usually used. Through the study of the bladder LCNEC genome, it may be one of the future development directions to explore the identification of early molecular markers and new targeted therapies, so as to improve diagnostic methods and multi-mode therapy, and achieve the purpose of improving prognosis.

## Data availability statement

The original contributions presented in the study are included in the article/**Supplementary Material**. Further inquiries can be directed to the corresponding author.

## Ethics statement

Written informed consent was obtained from the individual(s) for the publication of any potentially identifiable images or data included in this article.

## Author contributions

JC: Data curation, Investigation, Writing – original draft, Writing – review & editing. QZ: Writing – original draft. CY: Data curation, Investigation, Software, Writing – review & editing. PM: Data curation, Formal analysis, Methodology, Writing – review & editing. SL: Data curation, Formal analysis, Methodology, Writing – review & editing.
